# Analgesia in the Neurosurgical Intensive Care Unit

**DOI:** 10.3389/fneur.2021.819613

**Published:** 2022-01-25

**Authors:** Slavica Kvolik, Nenad Koruga, Sonja Skiljic

**Affiliations:** ^1^Faculty of Medicine, Josip Juraj Strossmayer University of Osijek, Osijek, Croatia; ^2^Department of Anesthesiology and Critical Care, Osijek University Hospital, Osijek, Croatia; ^3^Department of Neurosurgery, Osijek University Hospital, Osijek, Croatia

**Keywords:** analgesia, pain, intensive care units, neurosurgery, opioids, drug side effects, constipation, gastroparesis

## Abstract

Acute pain in neurosurgical patients is an important issue. Opioids are the most used for pain treatment in the neurosurgical ICU. Potential side effects of opioid use such as oversedation, respiratory depression, hypercapnia, worsening intracranial pressure, nausea, and vomiting may be problems and could interfere with neurologic assessment. Consequently, reducing opioids and use of non-opioid analgesics and adjuvants (N-methyl-D-aspartate antagonists, α2 -adrenergic agonists, anticonvulsants, corticosteroids), as well as non-pharmacological therapies were introduced as a part of a multimodal regimen. Local and regional anesthesia is effective in opioid reduction during the early postoperative period. Among non-opioid agents, acetaminophen and non-steroidal anti-inflammatory drugs are used frequently. Adverse events associated with opioid use in neurosurgical patients are discussed. Larger controlled studies are needed to find optimal pain management tailored to neurologically impaired neurosurgical patients.

## Introduction

Acute pain following acute brain injury could be substantial, but commonly it is not the primary consideration of the neurosurgical intensive care (NSICU) patients. The main reason for this is a priority of preventing secondary brain damage after trauma or surgical procedure (1, 2). Preservation of adequate cerebral perfusion and oxygenation while managing systemic intracranial pressure, mechanical ventilation, stabilization of circulation, fluid balance, temperature, and glycemic control in neurosurgical patients is complex and demanding but adequate pain control could improve outcome and patient satisfaction (3).

Several ERAS protocols (enhanced recovery after surgery) were recently developed for postoperative patients and perioperative pain treatment is included in them (4). Reduced preoperative fasting, maltodextrin fructose solution 2 h preoperatively, local infiltration of the surgical incision, and non-opioid analgesia are some of the maneuvers that were proven as efficient in awake patients (5). In addition to faster mobilization, these procedures also aim to reduce the use of opioids in the perioperative period, the side effect of which may be chronic opioid use and opioid dependence (6). It is usually easy to assess pain and administer appropriate pain medications in conscious patients, but it could be challenging in sedated, drowsy, or non-cooperative patients (7).

In this review, we will present the pain assessment and the most common modalities of analgesia in NSICU patients. We will discuss the problems associated with analgesia in patients with impaired consciousness, and complications of opioid use in patients with acute brain injury. Respiratory depression, gastrointestinal dysmotility, delirium, and addiction with possibilities to reduce them will be highlighted.

## Multimodal Analgesia Post-Craniotomy

Acute pain after craniotomy is considered moderate to severe during the first two postoperative days. It is often underrated and hard to estimate, potentially becoming chronic (8, 9). No analgesic regimen for post-craniotomy pain was proven as efficient for all patients (1, 10), although opioids provided superior acute pain relief compared to other drugs in small clinical studies (11). Despite their efficiency in pain control, potential side effects such as (over) sedation, respiratory depression, hypercapnia with worsening intracranial pressure, nausea, and vomiting may be problematic in neurosurgical patients and can interfere with neurologic assessment (12). The risk of opioid dependence limits safe opioid use for only a few days.

The introduction of the ERAS protocol in neurosurgery aims to accelerate postoperative recovery and length of stay in the ICU, and analgesia with fewer opioids in the early post-craniotomy period is crucial in achieving these goals. By adhering to these protocols, Elayat and co-workers reduced the length of stay in the ICU, reduced the number of episodes with VAS >4, and reduced opioid consumption in the patients undergoing elective craniotomy for supratentorial neurosurgery (5). Consequently, non-opioid analgesics as a part of a multimodal regimen with fewer side effects became standard of care in the postoperative pain treatment in the NSICU (3).

Local and regional anesthesia techniques, such as local scalp infiltration/block seem to be effective in the early postoperative period (13). Postoperative scalp infiltration was associated with a significant reduction in pain scores and the reduction of the opioid requirement over the 24 h postoperatively. No adverse events of the regional techniques were reported in a meta-analysis of the 7 RCTs with a total of 320 patients (13).

Among non-opioid agents, acetaminophen and non-steroidal anti-inflammatory drugs are used most frequently in the first postoperative days to reduce the dose of opioids (11, 14). Recently various adjuvants were added as a part of preemptive, intraoperative and early postoperative analgesia for craniotomy pain. These include non-steroidal analgesics: N-methyl-D-aspartate (NMDA) antagonists (ketamine), α2 -adrenergic agonists (dexmedetomidine) (10, 15, 16), as well as anticonvulsants (gabapentin and pregabalin) (17), corticosteroids, an intravenous local anesthetic (lidocaine), and non-pharmacological interventions, such as multipoint electro-acupuncture (3, 10, 11).

Analgesia in NSICU patients with acute brain injury must not be a simple extension of post-craniotomy analgesia.

## Pain Assessment Tools and Patients With Altered Mental Status

Pain treatment in patients who have altered mental status before and after craniotomy is challenging. In NSICU patients, pain may be postoperative, e.g., after a craniotomy. It can be caused by intubation, mechanical ventilation, insufficient mobilization, as well as placement of nasogastric tube, urinary catheter, or central venous catheter. Several factors including sepsis, use of vasopressors, multiple comorbidities, previous (18), or new neurological deficiencies may influence pain assessment and treatment (19).

There are several tools adopted for the pain assessment in mechanically ventilated ICU patients, and patients with altered mental status. Behavioral Pain Scale (BPS), Critical Care Pain Observation Tool (CPOT) (20), or Nociception Coma Scale-Revised (NCS-R) (21) are some of the tools widely used. The parameters evaluated by these tools are physiologic parameters, verbal response, motor response, or facial mimic, which may be irreversibly impaired by brain damage. A study conducted by Nazari and coworkers showed that most of the behaviors that have been observed during painful stimulation in patients with traumatic brain injury included facial expressions, sudden eye-opening, frowning, lip changes, clear movements of extremities, neck stiffness, and sighing or moaning (22). Using these tools may be helpful, but also misleading in patients with brain edema after hypoxic brain injury, or in patients with intracranial hemorrhage and hypertension.

Severgnini et coworkers compared CPOT and BPS to examine their applicability in awake and sedated patients. They confirmed that both scales correlate with pain. In the comparison, CPOT was more sensitive (BPS 62.7%, CPOT 76.5%), and BPS was found to be more specific (BPS 91.7%, CPOT 70.8%) for pain assessment. The best results were obtained by combining both scales (23). In another study, Ribeiro and coworkers have studied psychometric properties of the BPS in traumatic brain injury. They found that BPS had good internal consistency, good discriminant validity, moderate to excellent reliability, and high responsiveness, but also that deep sedation affected the increase of grading during painful procedures (24).

Currently, none of the above rating scales can be considered the gold standard for pain assessment in neurocritical patients. As with other ICU patients, pain should be routinely assessed using validated behavioral scales and documented, especially before invasive procedures and physical therapy (24).

Implementation of standardized pain assessment protocols for the NSICU patients is important, but the actual implementation and adherence to these protocols remains an area for improvement and further investigations. Vital signs should not be used as a sole measurement, and surrogates for pain assessment (24). Using these tools may be helpful and misleading in patients with brain edema after hypoxic brain injury, or in patients with intracranial hemorrhage and hypertension.

The implementation of clinical assessment tools may lead to reduced use of analgesic and sedative agents, as observed in the nurses' study carried by Gelinas and coworkers (25). In the study of Phillips and coworkers, the implementation of the CPOT led to increased frequency of the pain assessment and a significant increase in the administration of paracetamol, opiates, propofol, patient-controlled analgesia, modified-release opiates, and neuropathic pain agents (26). A similar observation that implementation of pain assessment tools led to the more liberal administration of opioid-based pain relief was observed in Mascarenhas' study. Nurses in their study have increased opioid administration by 100% (27). The administration of opioids may decrease the pain experience, but may increase respiratory depression, have an impact on alertness and vigilance, and may impair neuropsychological evaluation.

The situation is more complicated when patients' condition dictates prolonged sedation, mechanical ventilation, and when other invasive and painful procedures are routinely performed in NSICU. In such situations, a protocol called “analgesia first sedation” may be useful by increasing compliance with the ventilator but may lead to increased opioid use (27). In neurologically impaired patients, it may mask their neurologic assessment.

## Acute Brain Injury and Pain Control

In patients with acute brain injury, intracranial pressure oscillations are associated with hemodynamic instability and are treated with a reduction in intracranial pressure by continuous use of sedatives, such as propofol and opiates. Sedatives and opioids are given commonly, based on the blood pressure and pulse values to maintain smooth circulation and mechanical ventilation (28). Different opioids may be used like fentanyl, sufentanil, remifentanil, and morphine. Opioids are beneficial for analgesia, but their bolus administration may increase ICP with associated decreases in MAP and CPP, and should be avoided (29). In the systematic review Wiener and coworkers have found no consistent results between different opioids, and between opioid and non-opioid analgesia in the management of traumatic brain injury regarding their effects on MAP, ICP, or CPP (29). Opioid consumption can be reduced by the use of dexmedetomidine for sedation without affecting neurological function (30).

### Paroxysmal Sympathetic Hyperactivity

Paroxysmal sympathetic hyperactivity (PSH) is a syndrome resulting usually from traumatic brain injury (79.4% of the patients), and rarely from hypoxia (9.7%) or cerebrovascular accident (5.4%) (31). Since these conditions are commonly accompanied by severe brain swelling or blood extravasation, pain could be very strong although it is rarely reported and/or observed. PSH is characterized by simultaneous, paroxysmal transient increases in sympathetic (elevated heart rate, blood pressure, respiratory rate, temperature, sweating) and motor activity (31). Analgesia is particularly demanding in patients with impaired mental status and in those who have developed PSH (18). In patients with PSH, these symptoms may resemble withdrawal syndrome or acute pain (Table 1) and are very often treated with opioids (32–34). Until now, there are no studies reporting pain scores using any of the pain assessment tools in PSH patients.

**Table 1:** The most common symptoms of paroxysmal sympathetic hyperactivity, withdrawal syndrome, and acute pain in neurosurgical patients.

	**Paroxysmal sympathetic hyperactivity**	**Opioid withdrawal syndrome**	**Acute pain**
Tachycardia	++	++	++
Hypertension	++	++	++
Hyperventilation	++	+	+
Fever	++	+ (or hypothermia)	−
Profuse sweating	++	+	++
Restlesness	+	++	+
Muscle rigidity and hypertonus	++	Movement disorders, tremor	+/−
Agitation, insomnia	−	++	+
Other symptoms	Typical posturing	Rhinorrhoea, lacrimation, nausea, vomiting, diarrhea	↑ ICP

*Note: ++ present, + sometimes present, +/− not always present, − not registered, ↑ increase*.

Common problems with PSH are difficult mechanical ventilation, difficult maintenance of fluid and electrolyte balance, management of increased body temperature, increased number of blood specimens, and unresponsiveness of the symptoms to treatment.

One of the treatment goals in brain-injured patients is symptoms prevention and reduction (15). The symptoms of PSH are usually treated with various drugs, including morphine, non-selective β-blockers, short-acting benzodiazepines, baclofen, clonidine, non-steroidal anti-inflammatory drugs, α2 agonists, and GABA agonists. Although opioids and benzodiazepines are capable to control breakthrough episodes, due to their sedative effects and the possibility of addiction, other drug classes are gaining more popularity (35).

Currently, dexmedetomidine and gabapentin are used for both symptom and opioid reduction. In a recent study, Peng and coworkers have confirmed that dexmedetomidine reduces paroxysmal hypertension, average time for normalization of body temperature, heart rate, and respiratory rate below 25 breaths per minute, but does not protect against the recurrence of PSH (36). In the patients with severe traumatic brain injury and symptoms of paroxysmal sympathetic hyperactivity who underwent surgery, dexmedetomidine was not capable of either reducing ICU and hospital stay or of improving Glasgow outcome score (37).

Gabapentin is a promising drug for the treatment of PSH. Godo and colleagues reported a case series of patients who were resistant to benzodiazepines, opioids, and non-steroidal anti-inflammatory drugs. Initial gabapentin doses of 200 mg 3 times a day increased to 400 mg 3 times a day resulted in a reduction of PSH symptoms (32). To achieve wider use of gabapentin for PSH treatment, its effects observed in the single institution must be confirmed in a larger placebo-controlled study.

## Adverse Events of Opioids in Neurosurgical Patients

Although some preclinical studies have suggested that opioid receptor agonists may be beneficial in brain injury, capable of reducing brain edema as well as of providing neuroprotection during a stroke (38), these preclinical observations were not confirmed in clinical studies. Moreover, the use of opioids as an analgesic regimen in neurosurgical patients is associated with several clinical problems (side effects) such as oversedation, respiratory depression, prolonged mechanical ventilation, truncal rigidity, inappropriate immune modulation, development of opioid tolerance, opioid-induced hyperalgesia, ICU addiction, and delirium (39). Opioids also have direct cardiovascular effects, decreasing blood pressure, causing vasodilation, and decreasing cardiac work (40). Gastrointestinal-related side effects, which include constipation, nausea, vomiting, dry mouth, gastro-esophageal reflux, abdominal cramping, spasms, and bloating, are well-known as opioid-induced bowel dysfunction. Opioid-induced constipation (OIC) in patients receiving opioids is persistent and the most frequently reported side effect (41, 42).

### Respiratory Depression

Respiratory depression with consequent hypoxia and permanent brain injury has often been described after acute opioid intoxication (43). The outcomes of these patients are associated with the duration and severity of hypoxia. There are fewer studies correlating outcomes of the patients with acute brain trauma regarding opioid use and the duration of mechanical ventilation. Dexmedetomidine and propofol allow patients to wake up faster and breathe easier, although they are usually used with opioids in patients with acute brain injury. In the patients with acute brain injury, both dexmedetomidine and dexmedetomidine with propofol were associated with a significantly higher rate of hypotension as compared to propofol only or no sedation (44).

### Gastroparesis and Constipation

Gastrointestinal dysmotility, i.e., gastroparesis and bowel paralysis are commonly observed in the acute phase of injury in neurosurgical patients (45, 46). Both disorders are a complication of opioid use and brainstem lesions (Table 2). In 139 mechanically ventilated patients in general ICU, the incidence of opioid-induced constipation (OIC) was 63%, and gastric retention was 49% (53). Guerra and coworkers found OIC in 72% of critically ill patients (54). In their paper, by the study protocol patients on the parenteral nutrition therapy were excluded. The incidence of constipation would probably be even higher if such patients were accounted.

**Table 2:** Frequency of the most common gastrointestinal motility disorders in the neurosurgical intensive care unit and their association with risk factors.

**References**	**Postinjury phase**	**GI motility disorder (%)**	**Risk factors**
Lim et al. (47)	Acute poststroke	Constipation 39%	Immobility, bedpan use, a longer length of stay
Vieira et al. (48)	Traumatic brain injury- acute postinjury phase	Diarrhea 69.6%	Critical illness, enteral nutrition, antibiotics usage
Makkar et al. (49)	Traumatic brain injury- acute postinjury phase	Gastroparesis, gastric aspirate volume (GAV) 60.5%	Raised intracranial pressure, sympathetic stimulation, hyperglycemia, use of opioids.
Pinto et al. (45)	Traumatic brain injury- acute postinjury phase	Feeding intolerance 75.0%	Brain-gut dysfunction
Berry et al. (50)	Traumatic brain injury- acute postinjury phase	No bowel movement between 48 and 72 h 45.6%	Autonomic disturbances, hyper-sympathetic response, damage of hypothalamus, narcotic analgesics
Cai et al. (51)	Acute recovery sequelae phase	Constipation 41.6% Constipation 31.5 % Constipation 22.6%	Incidence higher with acute phase, basal ganglia inclusion, and cerebral hemorrhage
Robain et al. (52)	Rehabilitation after recent vascular hemiplegia	Constipation 60%	Brain lesions, functional status of patients (assessed by Barthel Index)
Cheng et al. (46)	Chronic	Constipation	Brainstem lesions, the desire for defecation threshold, physical activity level

Kieninger and coworkers studied a promotility protocol in adult NSICU patients with acute severe brain injury and anticipated mechanical ventilation for more than 3 days (55). All patients received sufentanil and sedation. Promotility procedures were colon massage, physical therapy, and early peroral lactulose, followed by naloxone 4 mg p.o. 3x/day. In the study group, an adequate defecation pattern was observed in 9 out of 37 (24.3%) compared to only 9 out of 109 (8.3%) in the control group (55). Opioid antagonists are considered a logical approach to treat OIC. In a recent study, both enteral naloxone and subcutaneous methylnaltrexone were effective for the treatment of OIC in medical ICU. The median times to first bowel movement were 30 and 24 h for naloxone and methylnaltrexone patients, respectively (56).

Schmittner and coworkers reported no difference in the time period until full enteral nutrition or first defecation between patients receiving opioid fentanyl and S(+)-ketamine in neurosurgical patients (57). This suggests that brain damage, *per se*, may influence gut dysfunction via unknown mechanisms. Larger prospective studies are needed to answer the true OIC incidence and optimal treatment in NSICU patients with brain injury.

### Hypotension

A decrease in the mean arterial blood pressure is well-known during fentanyl, remifentanil, or alfentanil administration (58), as well as during prolonged sedation in mechanically ventilated patients and it is accompanied by increased use of vasopressors in the medical ICU (59). The total amount of propofol and fentanyl correlated with vasopressor use and prolonged sedation. Patients who underwent analgesia with S(+)-ketamine showed a trend to lower demand for norepinephrine compared with the fentanyl group without an increase of ICP and CPP (57).

### Addiction in the ICU

Addicted patients are a growing category in ICU. Analyzing toxicological samples taken from 44 patients admitted to the Intensive Care Unit Ruiz-García and coworkers confirmed the use of ≥1 substance in 74% of patients. The most consumed substances were alcohol and tobacco (> 55%), and cannabis, amphetamines (> 11%), and cocaine (9%). Discontinuation of each of these substances can lead to withdrawal syndrome (60).

Opioid addiction is reflected in an individual pathologically pursuing reward and/or relief by substance use and other behaviors. Usually, they need a special approach while on opioid maintenance treatment during the ICU stay, since the opioid restriction is not recommended during the acute illness (61). The toxic effect of opioids and other substances of abuse may lead to ischemic brain damage and ICU admission in youth and younger adults (62). Non-traumatic hypoxic brain injury was observed in a total of 8% of the patients admitted into the ICU with opioid overdose with mortality of 10% (63). In the period after hypoxic brain injury, during the recovery in ICU, these patients may develop symptoms of withdrawal syndrome (Table 1) (64).

Withdrawal syndrome may be a serious complication during ICU treatment (33). The most severe symptoms can be experienced in patients who have discontinued opioid therapy at the NSICU, regardless of whether the patient was taking opioids before admission to the NSICU, or opioid therapy as part of analgesia during NSICU treatment. Risk factors associated with iatrogenic withdrawal syndrome include duration of therapy and cumulative drug dose (65). Critically ill patients with preexisting cognitive or functional impairment are at special risk to develop opioid withdrawal syndrome (66).

In NSICU where opioids are commonly used, withdrawal may be difficult to recognize. Its recognition could be even poorer because no valid tools were developed for ICU patients. Opioid withdrawal syndrome may mimic PSH or other manifestations of brain damage (Table 1). Opioid doses should be kept at a minimum, and non-opioid drugs are preferred, whenever their use is effective. There are several strategies for opioid weaning, including scheduled enteral opioids after continuous opioid infusions (66) and the use of non-opioid drugs like dexmedetomidine and clonidine.

Promoting non-opioid analgesics and reducing opioid use is important to minimize the use of opioids in previously opioid-naïve patients after discharge from the NSICU. To date, there are no studies to confirm the frequency of opioid use in neurosurgical patients after discharge from the ICU. Data from other fields of intensive care confirm that this proportion is significant. Based on the recent cohort studies 40.8% (346 in 848) medical ICU patients and 45% (32 of the 71 surgical) opiate-naïve patients were discharged with a new opioid prescription (67, 68). A shorter inpatient opioid therapy decreased the risk of post-discharge opioid therapy (68).

In clinical situations where opioid use cannot be avoided, nor can opioids be replaced by other drugs, opioid rotation may be useful. It can reduce both risks of opioid dependence and of opioid tolerance, i.e., increase the opioid dose to maintain equianalgesic effects (39, 69).

### ICU Delirium

Patients may develop delirium as a consequence of opioid administration in the ICU. Pisani and coworkers reported patients who received opioids had a longer duration of delirium (70). The incidence of delirium in neurosurgical patients may be as high as 42.2% as observed by Wang and coworkers (71), but a correlation between delirium and opioid use was not analyzed in the study. Opioid use in NSICU is a modifiable risk factor for delirium.

## Unanswered Questions in the Field

There are numerous unresolved questions in the field of analgesic use in acute brain injury, such as whether the choice of analgesics may influence neurological recovery in acute brain injury. In the *in vivo* study tramadol was able to minimize perivascular edema, neuronal necrosis, inflammatory cell infiltration in acute and chronic ischemia/reperfusion injury (72). The comparable neuroprotective effect was observed with dexmedetomidine in *in vitro* and *in vivo* studies. Possible mechanisms are signaling pathways for inflammatory response, oxidative stress, neurotransmitter regulation, mitochondrial function, apoptotic pathway, and autophagy (73). This effect was not confirmed in human studies. It is also unknown whether opioid rotation can reduce the incidence of ICU withdrawal syndrome, and post-discharge opioid use. It has been confirmed that the use of multimodal treatment under the ERAS protocol, such as the combination of non-opioid analgesics, and gabapentin, reduces opioid use in postoperative general surgical patients (74). While decreasing pain scores, it decreased the level of consciousness in a dose-dependent fashion, and prolonged stay in the post-anesthesia care unit, too (74). There are no such studies in the patients who suffered an acute brain injury. It is particularly unknown whether the neurological outcome may be modified in the patients who have received such a multimodal treatment. This should be confirmed by future studies.

## Conclusion

Both pain assessment and pain control are challenging in neurosurgical patients with altered consciousness. Opioid use is common in NSICU but can lead to respiratory depression and difficult neurological evaluation from oversedation. Avoiding opioids and the use of alternative medications and therapies are recommended. Multimodal postoperative analgesia and proper drug selection in the ICU may reduce the side effects of opioid treatment. Further studies should confirm whether the choice of analgesia and opioid restriction in patients with severe brain injury may influence outcomes.

## Author Contributions

SS and SK each wrote sections of the paper and performed the literature review. SK and NK have prepared tables for publication. SK, NK, and SS have approved a final version of the manuscript. All authors contributed to the article and approved the submitted version.

## Funding

This manuscript was supported by IP16-20 and IP1-21 by J.J. Strossmayer University, Medical Faculty, Osijek, Croatia.

## Conflict of Interest

The authors declare that the research was conducted in the absence of any commercial or financial relationships that could be construed as a potential conflict of interest.

## Publisher's Note

All claims expressed in this article are solely those of the authors and do not necessarily represent those of their affiliated organizations, or those of the publisher, the editors and the reviewers. Any product that may be evaluated in this article, or claim that may be made by its manufacturer, is not guaranteed or endorsed by the publisher.
